# Synergistic Utilization of Necrostatin-1 and Z-VAD-FMK Efficiently Promotes the Survival of Compression-Induced Nucleus Pulposus Cells via Alleviating Mitochondrial Dysfunction

**DOI:** 10.1155/2020/6976317

**Published:** 2020-12-08

**Authors:** Songfeng Chen, Qing Tian, Chunfeng Shang, Lin Yang, Na Wei, Guowei Shang, Yanhui Ji, Hongwei Kou, Shitao Lu, Hongjian Liu

**Affiliations:** ^1^Department of Orthopaedics, The First Affiliated Hospital of Zhengzhou University, Zhengzhou 450052, China; ^2^Department of Paediatrics, The Zhengzhou Central Hospital Affiliated to Zhengzhou University, Zhengzhou 450007, China; ^3^Department of Pathology, The First Affiliated Hospital of Zhengzhou University, Zhengzhou 450052, China

## Abstract

We recently reported that necroptosis contributed to compression-induced nucleus pulposus (NP) cells death. In the current study, we investigated the regulative effect of necroptosis inhibitor Necrostatin-1 on NP cells apoptosis and autophagy. Necrostatin-1, autophagy inhibitor 3-Methyladenine and apoptosis inhibitor Z-VAD-FMK were employed, and NP cells were exposed to 1.0 MPa compression for 0, 24 and 36 h. Necroptosis-associated molecules were measured by Western blot and RT-PCR. Autophagy and apoptosis levels were evaluated by Western blot and quantified by flow cytometry after monodansylcadaverine and Annexin V-FITC/propidium iodide staining, respectively. The cell viability and cell death were also examined. Furthermore, we measured mitochondrial membrane potential (MMP), mitochondrial permeability transition pore (MPTP) and indices of oxidative stress to assess mitochondrial dysfunction. The results established that Necrostatin-1 blocked NP cells autophagy, and 3-Methyladenine had little influence on NP cells necroptosis. The Necrostatin-1+3-Methyladenine treatment exerted almost the same role as Necrostatin-1 in reducing NP cells death. Necrostatin-1 restrained NP cells apoptosis, while Z-VAD-FMK enhanced NP cells necroptosis. The Necrostatin-1+Z-VAD-FMK treatment provided more prominent role in blocking NP cells death compared with Necrostatin-1, consistent with increased MMP, reduced opening of MPTP and oxidative stress. In summary, the synergistic utilization of Necrostatin-1 and Z-VAD-FMK is a very worthwhile solution in preventing compression-mediated NP cells death, which might be largely attributed to restored mitochondrial function.

## 1. Introduction

Low back pain is an important cause of disability worldwide [1, 2], which is strongly linked with intervertebral disc (IVD) degeneration [3, 4]. Many factors could lead to IVD degeneration, including aging, nutritional deficiency, and mechanical stimulation, in which mechanical compression is generally considered as a critical pathogenic factor [5, 6]. More and more researches focus on exploring compression-mediated nucleus pulposus (NP) cells death because these cells play a key role in the production of collagen II and aggrecan, which contribute in maintaining IVD homeostasis [7, 8].

The decrease in NP cells number is largely attributed to the degree of programmed cell death (PCD) [9]. For decades, apoptosis and autophagy, which were known as type I and II PCD, respectively, were viewed as the only two forms of regulated cell death [10]. Apoptosis is generally characterized by apoptotic body formation, intact plasma membrane, chromosome condensation, and caspase activation [11]. Autophagy is a “self-eating” process for maintaining cellular homeostasis, in which the injured proteins or organelles are encased in autophagic vesicles with bilayer membrane structure and then degraded for recycling [12]. Remarkably, necroptosis, also termed type III PCD, has received great attention in recent years [13, 14]. Unlike apoptosis, it is a caspase-independent mode of death. The initiation and execution of necroptosis are largely dependent on the activation of the receptor-interacting protein kinase 1 (RIPK1)/receptor-interacting protein kinase 3 (RIPK3)/mixed lineage kinase domain-like (MLKL) signaling pathway in most cases [13, 14].

Our latest studies reported that necroptosis inhibitor Necrostatin-1 exerted important protective role on compression-treated NP cells [15, 16]. Treatment with Necrostatin-1 in a singular manner efficiently protected against compression-induced NP cells death. Literatures demonstrated that there exists an interaction between necroptosis and autophagy; meanwhile, necroptosis and apoptosis pathways appear to be interrelated with each other under certain circumstances [12, 17]. However, the interactive effect between necroptosis and autophagy as well as necroptosis and apoptosis are intricate and mysterious [12, 17]. So systematically investigating the regulative effect of Necrostatin-1 on apoptosis and autophagy is expected to provide a more excellent strategy in reducing NP cells death during compression condition.

It has been well documented that mitochondrial dysfunction, which includes mitochondrial membrane potential (MMP) loss, ultrastructure disruption of mitochondria, enhanced opening of mitochondrial permeability transition pore (MPTP), overconsumption of adenosine-triphosphate (ATP), and overproduction of reactive oxygen species (ROS), is positively correlated to necroptosis, autophagy, and apoptosis [18, 19]. However, other literatures suggest that mitochondrial dysfunction is not closely related to necroptosis, autophagy, or apoptosis [20, 21]. Likewise, the exact mechanism of oxidative stress in compression-induced NP cells necroptosis, autophagy, and apoptosis has not been elucidated too.

In the current study, we investigated the mutual regulation between necroptosis and autophagy as well as necroptosis and apoptosis during compression-induced NP cells death. To gain a deeper understanding from the organelle level, we also interrogated the regulatory role of combined inhibition of the different PCDs on mitochondrial dysfunction of NP cells.

## 2. Materials and Methods

### 2.1. Isolation and Culture of Primary Rat NP Cells

All the animal experiments were performed in accordance with the protocol approved by the animal experimentation committee of the First Affiliated Hospital of Zhengzhou University. The 3-month-old Sprague-Dawley rats were purchased from the Experimental Animal Center of the First Affiliated Hospital of Zhengzhou University. We performed the rat NP cell isolation and culture as previously described [15, 16]. The second generation of NP cells was used in this study.

### 2.2. Compression and Pharmacological Treatment of Rat NP Cells

The model system was used as previously described, in which 1.0 MPa compression was loaded on NP cells to imitate *in vivo* condition [15, 16]. The cells were treated with DMSO (Control, Sigma, USA), necroptosis inhibitor Necronstatin-1 (Nec-1, Sigma, USA), autophagy inhibitor 3-Methyladenine (3-MA, Sigma, USA), and apoptosis inhibitor Z-VAD-FMK (Z-VAD, Merck, Germany) and then using a combination of the inhibitors: Nec-1+3-MA and Nec-1+Z-VAD. The bottom of the pressure vessel was filled with distilled water to preserve sufficient humidity and keep the device in an incubator at 37°C. 0 h mentioned in the experiment means the beginning of compression. The 0, 24, and 36 h compression-treated time points were selected in the current experiment according to our previous studies [15, 16].

### 2.3. Monodansylcadaverine (MDC) Staining

The autophagic vacuoles of NP cells were detected by MDC (Sigma, USA). At each time point, the cells were washed three times with PBS and incubated with 0.05 mM MDC solution for 15 min at 37°C in the dark. Finally, the intracellular MDC fluorescence was quantified under flow cytometry (BD LSRII, Becton Dickinson).

### 2.4. Determination of Cell Viability

NP cells were seeded into 96-well culture plates at a density of 5 × 10^3^ cells per well. 24 h later, the cells underwent 0, 24, or 36 h compression, and cell viability was evaluated using the CCK-8 detection kit (Dojindo, Japan) according to the manufacturer's instructions. The cell viability was quantified by absorbance detection at 450 nm with a spectrophotometer (BioTek, USA).

### 2.5. Lactate Dehydrogenase (LDH) Release

Following 0, 24, and 36 h compression, the release of LDH into the culture medium was measured to evaluate the cytotoxicity of NP cells using an automated chemistry analyzer as previously described (Beyotime, China). The LDH activity (reflecting cell death) was expressed as the percentage of LDH in the cell culture medium to total cellular LDH.

### 2.6. Annexin V-FITC and Propidium Iodide (PI) Positive Ratio

The Annexin V-FITC Apoptosis Detection Kit (Beyotime, China) was introduced to quantify apoptotic and necrotic ratio of NP cells. Following 0, 24, and 36 h compression, the cells were harvested, stained as previously described [15, 16], and analyzed using flow cytometry. The Annexin V-FITC and PI double staining was utilized to detect the apoptotic incidence (Annexin V ratio) of NP cells.

### 2.7. Evaluation of MMP

After 0, 24, and 36 h compression, the NP cells were labeled by fluorescence probe 5,5′,6,6′-tetrachloro-1,1′,3,3′-tetraethyl-benzimidazolylcarbocyanine iodide (JC-1, Keygen Biotech, China) as we previously described [15]. Finally, the samples were quantified by flow cytometry. The evaluation of MMP is expressed as the ratio of red to green fluorescence intensity.

### 2.8. Measurement of MPTP Opening

The MPTP opening of NP cells was assessed by the MPTP Assay Kit (Genmed, China) as previously described [15]. After 0, 24, and 36 h compression, the cells were collected; afterward, 500 *μ*l preheated cleaning solution (Reagent A) and isopyknic working solution containing neutralization and staining solution (Reagent B) were added into the cell suspension. Then, the above cell suspension was mixed gently and fully and incubated for 20 min at 37°C in the dark. Lastly, the samples were resuspended in Reagent A and analyzed by flow cytometry.

### 2.9. Measurement of ROS

Intracellular ROS of NP cells was examined by ROS-specific fluorescent probe 2′-7′-dihydrodichlorofluorescein diacetate (DCFH-DA, Sigma, USA). Briefly, following 0, 24, and 36 h compression, cells were stained with 10 *μ*M DCFH-DA for 30 min at 37°C in the dark. Then, the mean fluorescence intensity (MFI) was assayed by flow cytometry.

### 2.10. Mitochondrial ROS (mtROS) Analysis

The MitoSOX red (Merck, Germany), a live-cell permeant fluorescence dye for selective detection of superoxide in mitochondria, could emit red fluorescence after being oxidized by superoxide. Following 0, 24, and 36 h compression, the cells were incubated with 5 *μ*M MitoSOX red for 30 min at 37°C in the dark. Finally, the samples were washed three times, suspended in 200 *μ*l of PBS, and assayed via flow cytometry.

### 2.11. Measurement of MDA Content and SOD Activity

The MDA content of NP cells was detected using the Lipid Peroxidation MDA Assay Kit (Beyotime, China), and the SOD activity of NP cells was evaluated by the SOD Assay Kit (Beyotime, China). At each time point, the cells were lysed in lysis buffer and centrifuged at 12000 rpm for 15 min and the cell deposits were discarded. The supernatant was reacted with thiobarbituric acid (TBA), and then, the MDA content was analyzed via a spectrophotometer (BioTek, USA) at 532 nm. For SOD activity detection, the cells were collected, lysed, and centrifuged at 12000 rpm for 15 min. Then, the supernatant was obtained to evaluate SOD activity by a spectrophotometer. Finally, the MDA content was represented as nmol/mg protein and the SOD activity was represented as U/mg protein.

### 2.12. Western Blot Analysis

The NP cells were lysed in lysis buffer containing of 1% protease inhibitor. The protein concentration of lysate was determined using the enhanced BCA protein assay kit (Keygen Biotech, China). The whole lysate was separated by SDS polyacrylamide gel electrophoresis (SDS-PAGE) and then transferred onto polyvinylidene fluoride membranes. Membranes were blocked with 5% bovine serum albumin in TBST for 1 h at room temperature and then incubated overnight at 4°C with primary antibodies against RIPK1 (1 : 500, CST, USA), phospho-PKA substrate (1 : 1000, CST, USA), RIPK3 (1 : 500, Abcam, UK), pRIPK3 (phosphoS232, 1 : 1000, Abcam, UK), MLKL (1 : 500, Abcam, UK), LC3B (1 : 1000, Sigma, USA), Beclin1 (1 : 500, CST, USA), Cleaved Caspase-3 (1 : 500, Abcam, UK), Cleaved Caspase-8 (1 : 500, Proteintech, China), Cleaved Caspase-9 (1 : 200, Proteintech, China), and GAPDH (1 : 5000, Abcam, UK). After incubation, membranes were gently washed three times and incubated with respective peroxidase-conjugated secondary antibodies for 2 h at 4°C and washed again. Finally, the protein bands were developed and quantified by enhanced chemiluminescence procedure and normalized to GAPDH.

### 2.13. Quantitative Real-Time Polymerase Chain Reaction (qRT-PCR)

Total RNA was isolated from harvested NP cells using TRIzol reagent (Invitrogen, USA) according to the manufacturer's instructions. Then, the obtained RNA was transcribed into complementary DNA (cDNA). The primer sequences used for RT-PCR analysis were designed as follows: RIPK1: 5′-TCCTCGTTGACCGTGAC-3′, 5′-GCCTCCCTCTGCTTGTT-3′; RIPK3: 5′-CCAGCTCGTGCTCCTTGACT-3′, 5′-TTGCGGTCCTTG TAGGTTTG-3′; MLKL: 5′-TCTCCCAACATCCTGCGTAT-3′, 5′-TCCCGAGTGGTGTAACCTGTA-3′; and GAPDH: 5′-CGCTAACATCAAATGGGGTG-3′, 5′-TTGCTGACAATCTTGAGGGAG-3′. The RT-PCR analysis was performed via SYBR Green mix (ToYobo, Japan) in a Step One Plus Real-Time PCR System (Applied Biosystems, CA, USA). The gene expression was subjected to analysis of amplification curve, and the data was analyzed using the 2^-*ΔΔ*CT^ method and normalized to GAPDH.

### 2.14. Statistical Analysis

Numerical data were shown as mean ± standard deviation (SD) from at least three independent repetitive experiments. Statistical analyses were carried out using IBM SPSS software package 22.0. Multiple groups were analyzed by one-way analysis of variance (ANOVA), followed by Bonferroni's post hoc test. Student's *t* test was used to analyze the differences between the two groups. The difference was considered statistically significant when *P* < 0.05.

## 3. Results

### 3.1. Nec-1 Attenuates Compression-Induced NP Cells Autophagy

**Figure 1:**
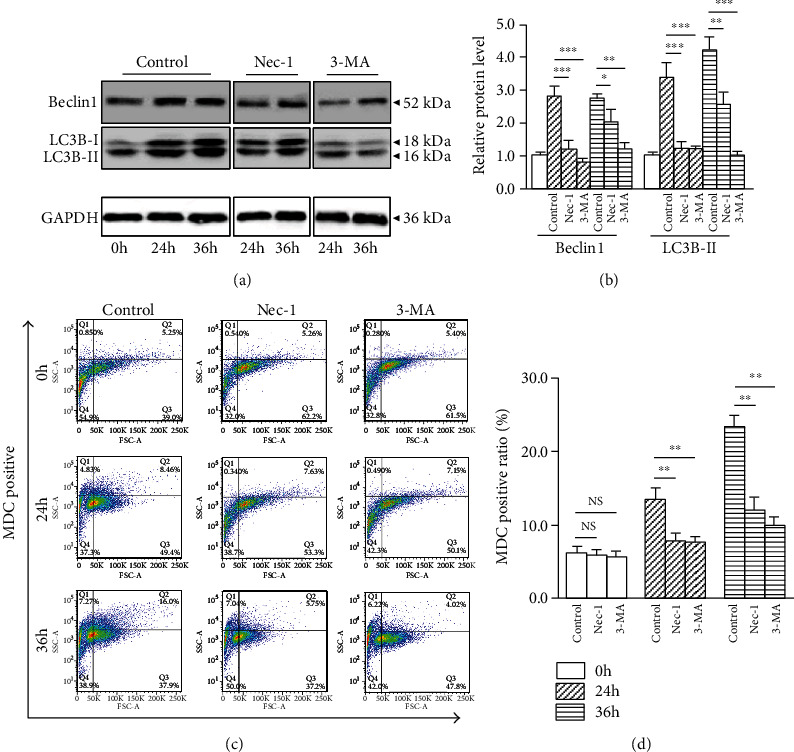
Nec-1 (20 *μ*M) or 3-MA (5 mM) attenuated 24 and 36 h compression-induced NP cells autophagy. (a, b) Western blot and quantitative analysis of autophagy-related molecules LC3B-II, Beclin1, and GAPDH in NP cells. (c, d) Representative dot plot images by flow cytometry after MDC staining and quantitative analysis of MDC positive ratio in NP cells. Data from treated groups have been normalized to GAPDH. NS means no significant statistical significance (^∗^*P* < 0.05, ^∗∗^*P* < 0.01, and ^∗∗∗^*P* < 0.001 vs. control).

To evaluate whether Nec-1 blocked NP cells autophagy, we measured the autophagy-associated molecules LC3B-II and Beclin1 expression. The 24 and 36 h compression provoked a distinguished upregulation expression of LC3B-II and Beclin1 compared with the 0 h group (Figures 1(a) and 1(b)). Treatment with 20 *μ*M Nec-1 or 5 mM 3-MA blocked the increased expression of LC3B-II and Beclin1 (Figures 1(a) and 1(b)). MDC labeling, which can be incorporated into lipids in autophagic vacuoles, was increased after 24 and 36 h compression (Figures 1(c) and 1(d)). Also, the Nec-1 or 3-MA treatment attenuated MDC positive ratio at 24 and 36 h (Figures 1(c) and 1(d)). These results implied that Nec-1 downregulated compression-induced NP cells autophagy.

### 3.2. 3-MA Has Little Influence on Compression-Induced NP Cells Necroptosis

**Figure 2:**
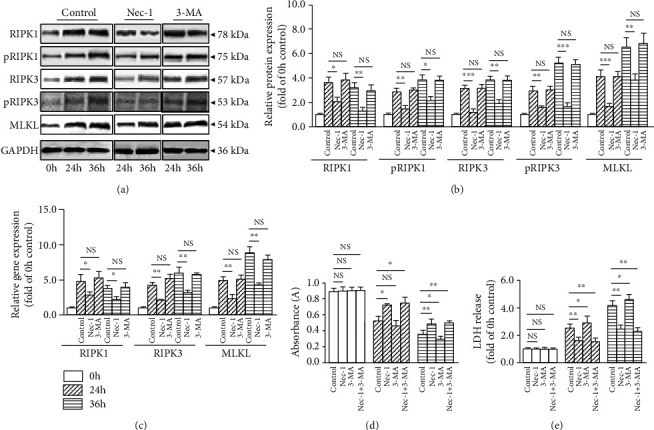
3-MA (5 mM) had little effect on NP cells necroptosis, and Nec-1+3-MA achieved almost the same effect as Nec-1 treatment in preventing compression-induced NP cells death. (a, b) Western blot and quantitative analysis of necroptosis-related molecules RIPK1, pRIPK1, RIPK3, pRIPK3, MLKL, and GAPDH in NP cells. (c) RT-PCR measured the mRNA expression of necroptosis-related genes RIPK1, RIPK3, MLKL, and GAPDH in NP cells. (d) CCK-8 assay showed the viability change of NP cells. (e) LDH release exhibited the cytotoxicity of NP cells. NS means no significant statistical significance (^∗^*P* < 0.05, ^∗∗^*P* < 0.01, and ^∗∗∗^*P* < 0.001 vs. control).

To explore the regulatory effect of 3-MA on NP cells necroptosis, the expression level of necroptosis-associated molecules RIPK1, pRIPK1, RIPK3, pRIPK3, and MLKL were measured by Western blot and RT-PCR. The results demonstrated that compared with 0 h, the protein expression of RIPK1, pRIPK1, RIPK3, pRIPK3, and MLKL as well as the gene expression of RIPK1, RIPK3, and MLKL significantly increased following 24 and 36 h compression (Figures 2(a)–2(c)). Treatment with Nec-1 reduced RIPK1, pRIPK1, RIPK3, pRIPK3 and MLKL expression at both 24 and 36 h (Figures 2(a)–2(c)). 3-MA had little influence on RIPK1, RIPK3 and MLKL in both protein and gene levels at 24 and 36 h; meanwhile, 3-MA had little effect on pRIPK1 and pRIPK3 too (Figures 2(a)–2(c)). Considering that Nec-1 blocked NP cells autophagy, we speculated that necroptosis might be an upstream mediator of autophagy.

### 3.3. Nec-1+3-MA Achieves Almost the Same Effect as Nec-1 in Protecting against Compression-Induced NP Cells Death

The cell viability and LDH release were examined to determine NP cells survival capability. After 24 and 36 h compression, Nec-1+3-MA or Nec-1 treatment increased NP cells viability compared with the control group. This beneficial effect was almost the same between the Nec-1+3-MA and Nec-1 group (Figure 2(d)). Likewise, Nec-1+3-MA or Nec-1 treatment exerted a more effective role than control in reducing LDH release at 24 and 36 h (Figure 2(e)). No apparent differences were observed between the Nec-1+3-MA and Nec-1 group. These results indicated that Nec-1+3-MA exerted almost the same effect as Nec-1 in preventing compression-induced NP cells death.

Hence, the following study mainly focuses on researching the mutual regulation between necroptosis and apoptosis and the synergistic inhibition of necroptosis and apoptosis on compression-induced NP cells death.

### 3.4. Nec-1 Blocks Compression-Induced NP Cells Apoptosis

**Figure 3:**
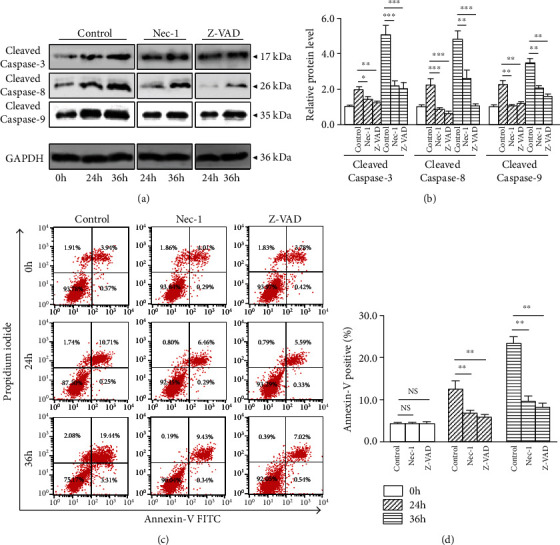
Nec-1 (20 *μ*M) or Z-VAD (20 mM) attenuated 24 and 36 h compression-induced NP cells apoptosis. (a, b) Western blot and quantitative analysis of apoptosis-related molecules Cleaved Caspase-3, Cleaved Caspase-8, and Cleaved Caspase-9 and GAPDH in NP cells. (c, d) Representative dot plot images of apoptosis NP cells by flow cytometry after Annexin V staining and quantitative analysis. Data from treated groups have been normalized to GAPDH. NS means no significant statistical significance (^∗^*P* < 0.05, ^∗∗^*P* < 0.01, and ^∗∗∗^*P* < 0.001 vs. control).

To assess whether Nec-1 restrained compression-induced NP cells apoptosis, the proapoptotic molecules Cleaved Caspases were measured. Compared with the 0 h group, 24 and 36 h compression increased Cleaved Caspase-3, Cleaved Caspase-8 and Cleaved Caspase-9 expression (Figures 3(a) and 3(b)). Nec-1 or Z-VAD prevented the upregulation of Cleaved Caspase-3, Cleaved Caspase-8 and Cleaved Caspase-9 at 24 and 36 h (Figures 3(a) and 3(b)). The Annexin V positive (apoptosis) ratio was increased after 24 and 36 h compression compared with 0 h (Figures 3(c) and 3(d)). The Nec-1 or Z-VAD treatment reduced Annexin V positive ratio at 24 and 36 h (Figures 3(c) and 3(d)). These results suggested that compression resulted in NP cells apoptosis time dependently, which was blocked by Nec-1 or Z-VAD.

### 3.5. Z-VAD Enhances Compression-Induced NP Cells Necroptosis

**Figure 4:**
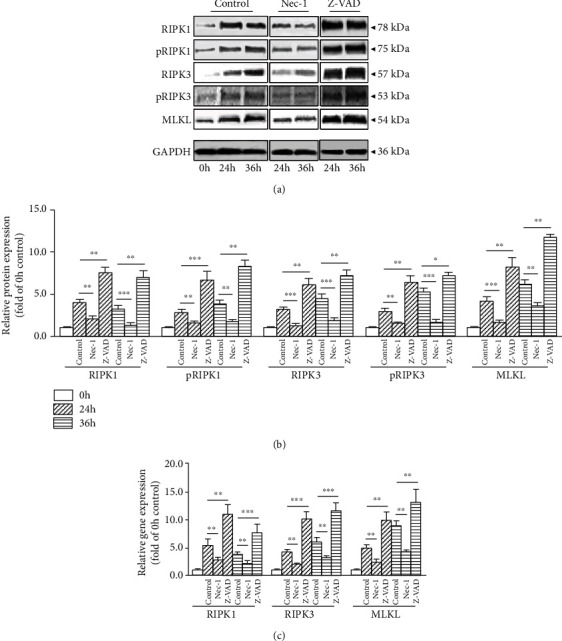
Z-VAD (20 mM) enhanced 24 and 36 h compression-induced NP cells necroptosis. (a, b) Western blot and quantitative analysis of necroptosis-related molecules RIPK1, pRIPK1, RIPK3, pRIPK3, MLKL, and GAPDH in NP cells. (c) RT-PCR measured the mRNA expression of necroptosis-related genes RIPK1, RIPK3, MLKL, and GAPDH in NP cells. Data from treated groups have been normalized to GAPDH (^∗^*P* < 0.05, ^∗∗^*P* < 0.01, and ^∗∗∗^*P* < 0.001 vs. control).

To explore whether Z-VAD could affect compression-induced NP cells necroptosis, the expression of RIPK1, pRIPK1, RIPK3, pRIPK3 and MLKL were measured. Different from 3-MA, a time course-related upregulation of RIPK1, pRIPK1, RIPK3, pRIPK3 and MLKL in the protein level and enhanced expression of RIPK1, RIPK3, and MLKL in the gene level were observed following Z-VAD treatment at 24 and 36 h (Figures 4(a)–4(c)). These results suggested that, under compression condition, blockage of apoptosis might result in partial conversion to necroptosis of NP cells.

### 3.6. Nec-1+Z-VAD Efficiently Protected against Compression-Mediated NP Cells Death

**Figure 5:**
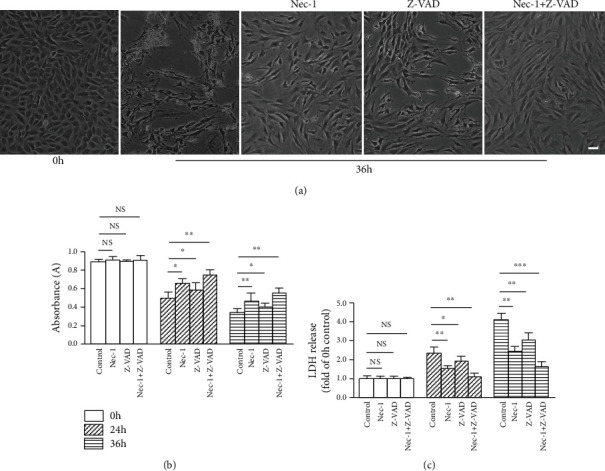
Nec-1+Z-VAD notably inhibited compression-induced NP cells death. (a) The morphological change of NP cells was observed by an optical microscope. (b) CCK-8 assay showed the viability change of NP cells. (c) LDH release exhibited the cytotoxicity of NP cells. Scale bars = 20 *μ*m. (^∗^*P* < 0.05, ^∗∗^*P* < 0.01, and ^∗∗∗^*P* < 0.001 vs. control).

There were no obvious differences in morphology of NP cells between 24 and 36 h time periods [16]. Thus, 36 h compression was chosen for morphology evaluation. Compared with 0 h, 36 h compression caused a majority of cells detaching from the culture plates and displaying morphological changes of necrosis (Figure 5(a)). The morphological observations indicated that Z-VAD offered mild protective effect. Simultaneously, Nec-1 or Nec-1+Z-VAD, especially the Nec-1+Z-VAD treatment, provided a remarkable protective role against compression-induced NP cells death (Figure 5(a)).

The cell viability and LDH release were examined to evaluate NP cells survival. After 24 and 36 h compression, Nec-1, Z-VAD, or Nec-1+Z-VAD treatment increased NP cells viability compared to the control group. This beneficial effect was more prominent following Nec-1+Z-VAD treatment (Figure 5(b)). Likewise, Nec-1+Z-VAD treatment exerted a more effective role than the Nec-1, Z-VAD, or control group in reducing LDH release at both 24 and 36 h time periods (Figure 5(c)). These results indicated that combined inhibition of necroptosis and apoptosis was more effective in protecting against compression-induced NP cells death than inhibition of necroptosis or apoptosis alone.

### 3.7. Nec-1+Z-VAD Rescues Compression-Mediated MMP Loss and MPTP Opening in NP Cells

**Figure 6:**
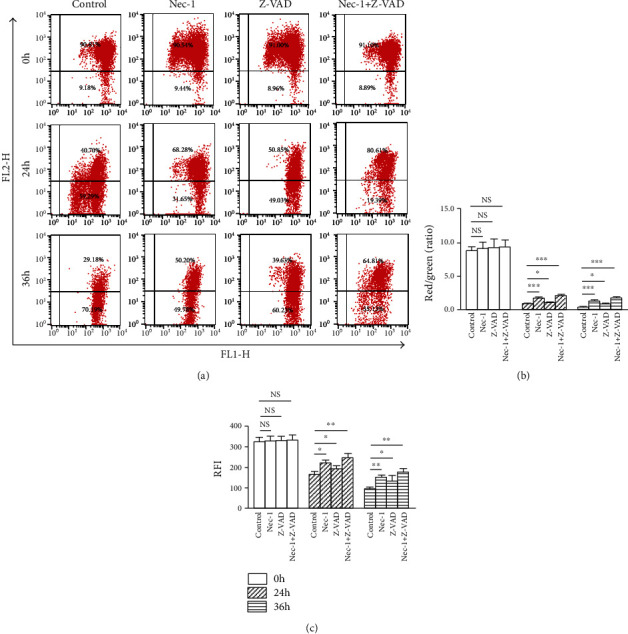
Nec-1+Z-VAD efficiently rescued 24 and 36 h compression-mediated MMP loss and MPTP opening in NP cells. (a) Representative dot plot images after JC-1 staining by flow cytometry in NP cells. (b) The quantitative analysis of JC-1 fluorescence intensity was expressed as the red/green ratio in NP cells. (c) The quantitative analysis of RFI of MPTP in NP cells by flow cytometry. NS means no significant statistical significance (^∗^*P* < 0.05, ^∗∗^*P* < 0.01, and ^∗∗∗^*P* < 0.001 vs. control).

The JC-1 aggregates (red fluorescence) are dispersed to monomeric form (green fluorescence) during the process of MMP loss, which could well reflect mitochondrial dysfunction. After 24 and 36 h compression, a time-dependent MMP loss occurred, as demonstrated by the decrease in red fluorescence and increase in green fluorescence compared with the 0 h group (Figures 6(a) and 6(b)). When treated with Nec-1, Z-VAD, or Nec-1+Z-VAD, especially the Nec-1+Z-VAD group, 24 and 36 h compression-stimulated MMP loss was efficiently rescued (Figures 6(a) and 6(b)). A significant feature of mitochondrial dysfunction is enhanced opening of MPTP. After 24 and 36 h compression, as shown in (Figure 6(c)), the relative fluorescence intensity (RFI) value of NP cells was gradually decreased, implying that enhanced MPTP opening occurred when compared with 0 h. In the presence of Nec-1, Z-VAD, or Nec-1+Z-VAD, especially in the Nec-1+Z-VAD group, 24 and 36 h compression-induced decrease of RFI was notably restored (Figure 6(c)). Taken together, these results implied that Nec-1+Z-VAD ameliorated NP cells injury via restraining excessive MPTP opening and MMP loss.

### 3.8. Nec-1+Z-VAD Alleviates Compression-Induced Oxidative Stress of NP Cells

**Figure 7:**
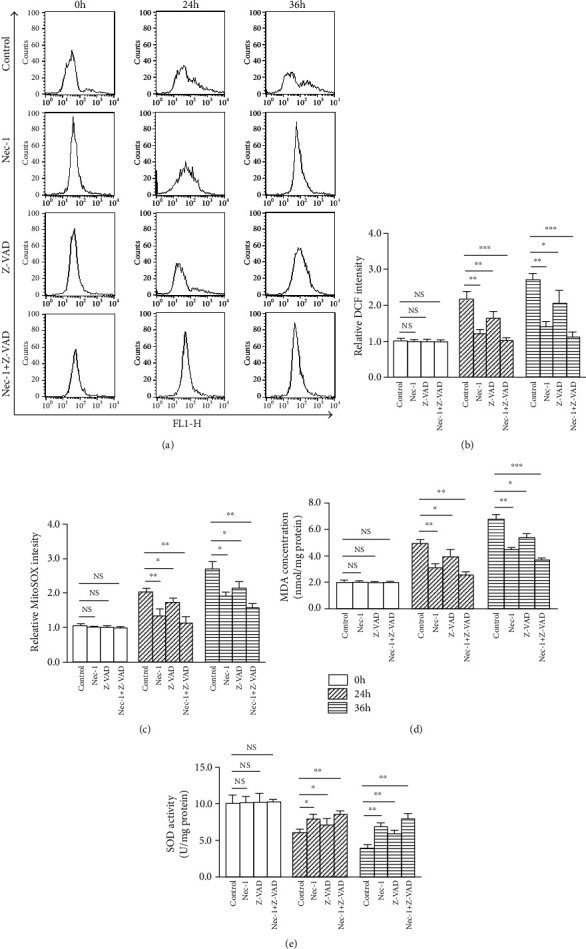
Nec-1+Z-VAD alleviated 24 and 36 h compression-provoked oxidative stress of NP cells. (a) Representative dot plot images after the labeling of fluorescent probe DCFH-DA in NP cells by flow cytometry. (b) The quantitative analysis of ROS in NP cells by flow cytometry. (c) The quantitative analysis of mtROS in NP cells by flow cytometry. (d) TBA method measured the content of MDA in NP cells. (e) The activity of SOD in NP cells was evaluated by spectrophotometry. NS means no significant statistical significance (^∗^*P* < 0.05, ^∗∗^*P* < 0.01, and ^∗∗∗^*P* < 0.001 vs. control).

Mitochondria have been generally considered as a crucial source of oxidative stress, and excessive activation of oxidative stress indirectly reflects mitochondrial dysfunction. Compared with the 0 h group, the ROS production (as indicated by DCFH-DA) was elevated after 24 and 36 h compression (Figures 7(a) and 7(b)). Similarly, an enhanced mtROS generation (MitoSOX fluorescence) was observed at both 24 and 36 h (Figure 7(c)). Treatment with Nec-1, Z-VAD, or Nec-1+Z-VAD, especially the Nec-1+Z-VAD treatment, reduced both the total ROS and mtROS fluorescence intensity (Figures 7(a)–7(c)).

MDA is positively correlated with oxidative stress damage, and SOD is an antioxidative enzyme. The MDA content was gradually increased while SOD activity was decreased in NP cells following 24 and 36 h compression (Figures 7(d) and 7(e)). Similar to ROS, treatment with Nec-1, Z-VAD, or Nec-1+Z-VAD, especially the Nec-1+Z-VAD group, notably blocked the upregulation of MDA content and downregulation of SOD activity at 24 and 36 h (Figures 7(d) and 7(e)). These results implied that Nec-1+Z-VAD attenuated compression-induced NP cells death might through alleviating oxidative stress.

## 4. Discussion

It has been well documented that a main contributor of IVD degeneration is NP cells death, which can be notably enhanced by compression [22, 23]. In the current study, we reported that autophagy was a downstream effect of necroptosis. Meanwhile, the mutual conversion between necroptosis and apoptosis might exist, and synergetic inhibition of necroptosis and apoptosis enables more efficient survival of NP cells compared with inhibition of necroptosis alone, which might be closely related with mitochondrial dysfunction-oxidative stress pathway.

Necroptosis is a brand-new type of PCD. Blockage of necroptosis inhibits compression-induced NP cells death [16]. As for the interactive effect between necroptosis and autophagy, there mainly exist three different views including upregulation of necroptosis following activation of autophagy [24], autophagy attenuating necroptosis [25], or autophagy as a downstream consequence of necroptosis [26]. Hence, the interaction between necroptosis and autophagy needs further study to clarify. In our preliminary experiments, we discovered that Nec-1 blocked compression-induced NP cells autophagy.

We focus on the interaction between necroptosis and autophagy of NP cells. The Nec-1 treatment prevented 24 and 36 h compression-mediated increase of LC3II and Beclin1. Nec-1 also attenuated compression-induced MDC positive ratio at 24 and 36 h. Meanwhile, 3-MA had little influence on RIPK1, RIPK3 and MLKL expression as well as RIPK1 and RIPK3 phosphorylation. Therefore, we concluded that necroptosis might be an upstream mediator of autophagy. Then, we explored whether combined inhibition of necroptosis and autophagy could efficiently protect against compression-induced NP cells death. The data indicated that Nec-1+3-MA exerted roughly the same effect as Nec-1 alone in preventing NP cells viability loss and cell death. These results are consistent with those of Lin et al., who confirm that autophagy is a downstream effect of necroptosis [26]. This might be partly attributed to the fact that RIPK1, the key promoter of necroptosis, could mediate the occurrence of autophagy too [27].

Hence, the following study mainly investigates the mutual regulative effect between necroptosis and apoptosis in NP cells. Literatures indicate that blockage of apoptosis enhances or decreases necroptosis, implying that inhibition of apoptosis could potentiate or attenuate the progression toward necroptosis [28, 29]. Furthermore, inhibition of necroptosis is reported to promote or decrease apoptosis too [30, 31]. Therefore, systematical elucidation of the “crosstalk effect” between necroptosis and apoptosis is expected to provide effective intervention targets for inhibiting NP cells death. We observed that Nec-1 obviously blocked Cleaved Caspase-3, Cleaved Caspase-8, and Cleaved Caspase-9 expression and Annexin V positive ratio in NP cells. Oppositely, when treated with Z-VAD, an enhanced expression of RIPK1, pRIPK1, RIPK3, pRIPK3 and MLKL were detected. That is to say, Nec-1 protected against compression-induced NP cells apoptosis and blockage of apoptosis resulted in partial conversion to necroptosis.

After 24 and 36 h compression, Nec-1+Z-VAD treatment achieved a more prominent effect in reducing NP cells death compared with Nec-1 or Z-VAD. Generally, Caspase-8 plays a switching role in the process of necroptosis and apoptosis. When Caspase-8 is activated, it could cleave RIPK3 and initiate apoptosis via formation of Complex IIa which contains RIPK1, RIPK3, Caspase-8, etc. However, when the Caspase-8 pathway is blocked or deleted, it could phosphorylate RIPK1 and RIPK3. The phosphorylated RIPK1 and RIPK3 then form Complex IIb (also termed necrosome) including RIPK1, RIPK3, and TNFR-associated death domain (TRADD), ultimately initiating necroptosis [32]. Therefore, we speculate that the protective role of Nec-1+Z-VAD is largely attributed to the blockage of the shunt between necroptosis and apoptosis.

Synergetic inhibition of necroptosis and apoptosis is indeed effective in inhibiting NP cells death. However, what is the underlying mechanism? Mitochondria have been recognized to have a critical role in cellular bioenergetics and redox [33, 34]; thus far, the precise mechanism of mitochondrial dysfunction in necroptosis or apoptosis remains elusive [18–21]. Generally, mitochondria are not only the major source of oxidative stress but also the vulnerable aim of oxidative stress [35]. The moderate activation of oxidative stress could promote cell survival; however, the overactivation of oxidative stress results in necroptosis, apoptosis, or cell death [36, 37]. Mitochondrial dysfunction and oxidative stress often interact with each other and synergistically determine the ultimate fate of cells [35, 38].

The contribution of combined inhibition of necroptosis and apoptosis to mitochondrial dysfunction and oxidative stress remains unclear in NP cells. It was displayed that 24 and 36 h compression provoked a time-dependent mitochondrial dysfunction. The Nec-1+Z-VAD treatment efficiently blocked MPTP opening and MMP loss in NP cells. Considering that enhanced MPTP opening directly results in mitochondrial injury, the protective role might be largely attributed to restrained opening of MPTP. Additionally, the ROS and mtROS production were remarkedly inhibited by Nec-1 or Nec-1+Z-VAD, especially in the Nec-1+Z-VAD group. The above results suggested that Nec-1+Z-VAD capably alleviates mitochondrial dysfunction and oxidative stress. The underlying mechanism might be similar to the protective effect of synergetic inhibition of necroptosis and apoptosis on NP cells survival, which was discussed above.

In conclusion, autophagy might be a downstream effect of necroptosis and the interaction between necroptosis and apoptosis existed in NP cells. Combined inhibition of necroptosis and apoptosis enables predominant effect on NP cells survival, which might be largely attributed to restored mitochondrial function. The synergistic utilization of Nec-1 and Z-VAD is a worthwhile strategy in reducing compression-induced NP cells death or even delaying IVD degeneration.

## Data Availability

The data in the manuscript have been repeated at least three times and are all available. The data can be accessed from Songfeng Chen (email: csfzdyfygk@163.com) and Hongjian Liu (email: fccliuhj@zzu.edu.cn).
